# Bio-acceptability of wearable sensors: a mechanistic study towards evaluating ionic leaching induced cellular inflammation

**DOI:** 10.1038/s41598-022-13810-0

**Published:** 2022-06-24

**Authors:** Pulak Bhushan, Vivek Kamat, Ishita Abrol, Ajeet Kaushik, Shekhar Bhansali

**Affiliations:** 1 Department of Electrical and Computer Engineering, Florida International University, 10555 W Flagler St. Miami, Florida, 33174 USA; 2 NanoBiotech Lab, Health System Engineering, Department of Environmental Engineering, Florida Polytechnic University, Lakeland, FL 33805-8531 USA

**Keywords:** Biomedical engineering, Electrical and electronic engineering, Sensors and probes, Apoptosis, Sensors, Electronic materials, Sensors and biosensors

## Abstract

The recent need for remote health wellness monitoring has led to the extensive use of wearable sensors. Owing to their increased use, these sensors are required to exhibit both functionality and safety to the user. A major component in the fabrication of these sensors and their associated circuitry is the use of metallic/organic conductive inks. However, very less is known about the interfacial and molecular interactions of these inks with biological matter as they can result in an inflammatory reaction to the user. Significant efforts are thus needed to explore and improve the bio-acceptability of such conductive ink-based wearable sensors. The present study investigates the biocompatibility of encapsulated and non-encapsulated wearable electrochemical sensors used for sensing uric acid as a biomarker for wound healing fabricated using screen-printing technique. Ionic release of metallic ions was investigated first to understand the susceptibility of the conductive inks towards ionic leaching when in contact with a fluid. Time-lapse investigation using ICPS (inductive couple plasma spectroscopy) shows a high concentration (607.31 ppb) of leached silver (Ag^+^) ions from the non-encapsulated sensors. The cell viability data suggests a 2.5-fold improvement in the sensor biocompatibility for an encapsulated sensor. While the carbon ink shows negligible effect on cell viability, the silver ink elicits significant decrease (< 50%) in cell viability at concentrations higher than 2 mg ml^-1^. The toxicity pathway of these sensors was further determined to be through the generation of reactive oxygen species resulting in over 20% apoptotic cell death. Our results show that the lower biocompatibility of the non-encapsulated sensor attributes to the higher leaching of Ag^+^ ions from the printed inks which elicits several different inflammatory pathways. This work highlights the importance biocompatibility evaluation of the material used in sensor fabrication to develop safe and sustainable sensors for long-term applications.

## Introduction

The recent escalation in the role of wearable devices for health and disease management has led to their usage for extended periods. Given the extensive usage, recent reports show that these devices can elicit inflammatory reactions and topical irritations^1^. A commonly reported side effect of continued use of wearable activity trackers is shown to be a skin rash^2^. Several research groups around the globe are working on identifying the potential causes for these reactions.Some studies attributed the induced inflammation to nickel leaching. The casings of most of these wearable devices are made of an alloy of stainless-steel containing nickel. The studies show that nickel can cause allergic contact dermatitis^3,4^, which can further spread to other parts of the body. Another study reported that the cause of localized contact dermatitis is the leaching of acrylate from the rechargeable battery housing^3^. The literature suggests that the management of inflammatory skin conditions triggered by wearable devices can be challenging. The present study thus attempts to understand the source of these inflammatory responses for the future development of biocompatible and sustainable devices.

Conductive inks have been extensively investigated for the development of wearable devices, such as physical or electrochemical sensors^5,6^, and flexible electronics^7–9^. Screen-printing of these conductive inks has been of particular interest owing to the techniques’ ease of fabrication and low cost. In contrast to other traditional fabrication routes that use conductive inks such as nanoimprint lithography^10,11^, and inkjet printing^12–14^ screen-printing allows for rapid fabrication and easy design customization, thus attracting a lot of attention^15,16^. Typically, these printed sensors interface directly with the user’s skin to acquire the required readings. These conductive inks are generally a heterogeneous mix of several constituent materials, some of which can induce an undesirable biological response if leached into the user’s body. Given that they are placed in direct contact with the skin, the user will be exposed to these materials primarily through the transdermal route. To further add to the problem, depending on the structural properties, certain materials have also been observed to cross the epithelial barrier of the skin and enter the blood circulation^17–19^. The translocation of these materials to secondary organs may result in widely distributed toxic effects. We thus hypothesize that material leaching through these conductive inks may be a potential cause for the reported inflammatory responses.

Two primarily used conductive materials for the development of such inks are carbon and silver. Several allotropes of carbon have been used for the synthesis of conductive inks^20,21^. Recent studies conducted to understand the potential toxic and immunological effects of carbon-based materials showed that their toxicity is dependent on several factors, such as their size and structure^22^. However, given the inherent biocompatible nature of the carbon-based materials used in these inks, the biocompatibility of carbon inks has been of little or no concern. On the other hand, owing to their high electrical and thermal conductivity, silver inks have widespread applications in the fabrication of wearable sensors and conductive traces in flexible electronics^23,24^. The biocompatibility of silver, especially in nanoparticle form, has always remained a concern, significantly limiting its practical usage. Silver shows toxicity towards a wide array of biological systems including mammalian cells, bacteria, and fungal cells^27^. While several studies^25–28^ have been conducted to understand its toxicity in various forms, the toxicity of silver leached through printed conductive inks is yet to be assessed. Silver nanoparticles are known to leach out from several products that contain silver when placed in contact with a fluid^29–31^. Our study hypothesizes that printed conductive silver inks will leach out silver ions when exposed to a (bio)fluid. Previous studies have shown that physicochemical properties of silver particles, e.g., particle size or surface charge, influence their toxic behavior. Several different studies have unanimously reported that smaller silver particles exhibit higher toxicity when compared to their larger counterparts^25,26^. Moreover, a study carried out by Smith et al., showed that silver ions elicit higher toxicity when compared to silver nanoparticles due to their smaller size and surface charge^32^. Also, the presence of other ions/metal complexes in the environment has been known to affect the toxicity profile^33,34^. One such study showed that the toxic response of silver ions can be mediated by the presence of chloride ions in the medium^35^. This study is especially interesting in the context of wearable electrochemical sensors since inks containing silver in combination with silver chloride are commonly used for the fabrication of reference electrodes. In addition to considering the effect of the leached particles’ properties and their environment, the overall packaging of the wearable device should also be accounted for. Seamless interfacing of wearable devices with the complex contours of the tissue requires high flexibility. Polymers are well suited for this purpose and have thus been widely used to encapsulate such wearable devices^36–38^. However certain polymers, for instance, isobornyl acrylate, can act as allergens, yet again resulting in an inflammatory skin response^39,40^. Assessment of the toxic response of polymer-encapsulated conductive inks is a knowledge gap that is yet to be addressed.

**Figure 1:**
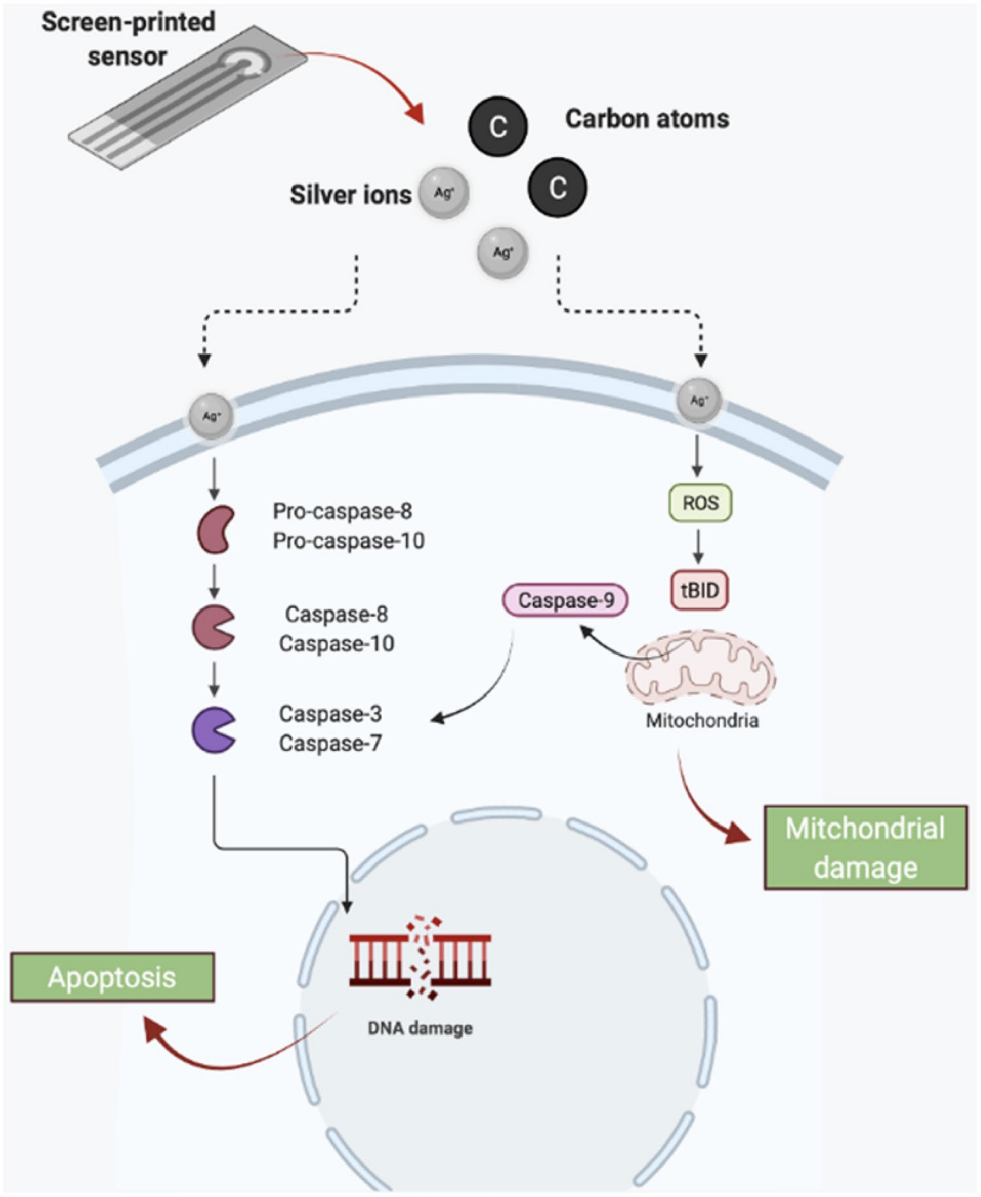
Schematic of wearable sensors interacting and eliciting biological response.

In this work, we explore the biocompatibility of two commercially available conductive inks, namely, carbon, and silver/silver chloride. As a proof of concept, a standard three-electrode electrochemical sensor, screen-printed on a flexible substrate using conductive inks, was selected for this study. The bio-acceptability assessment of the printed sensors was performed through in vitro cell assays. Since keratinocytes primarily constitute the human epidermis, it was used as the surrogate cell line in this study to investigate its response to the printed sensors. The study first aims to understand the effect of encapsulation on material leaching from the two printed inks through a mass spectrometric analysis. Following this, the toxicity profile of the differently encapsulated printed inks was investigated at cellular and intracellular level on an epidermal (HaCaT) cell line to identify the toxicity pathway of the leached particles. The interaction of silver and carbon ink with cells and their concentration-dependent changes in the cellular function, generation of reactive oxygen species, and apoptotic activity of the cells are detailed in this work (Fig. 1). A comprehensive understanding of the toxicity pathway of printed inks developed through this study can be used to inform a sustainable and biocompatible design pathway for future wearable devices.

## Results

### Assessment of ion leaching

**Figure 2:**
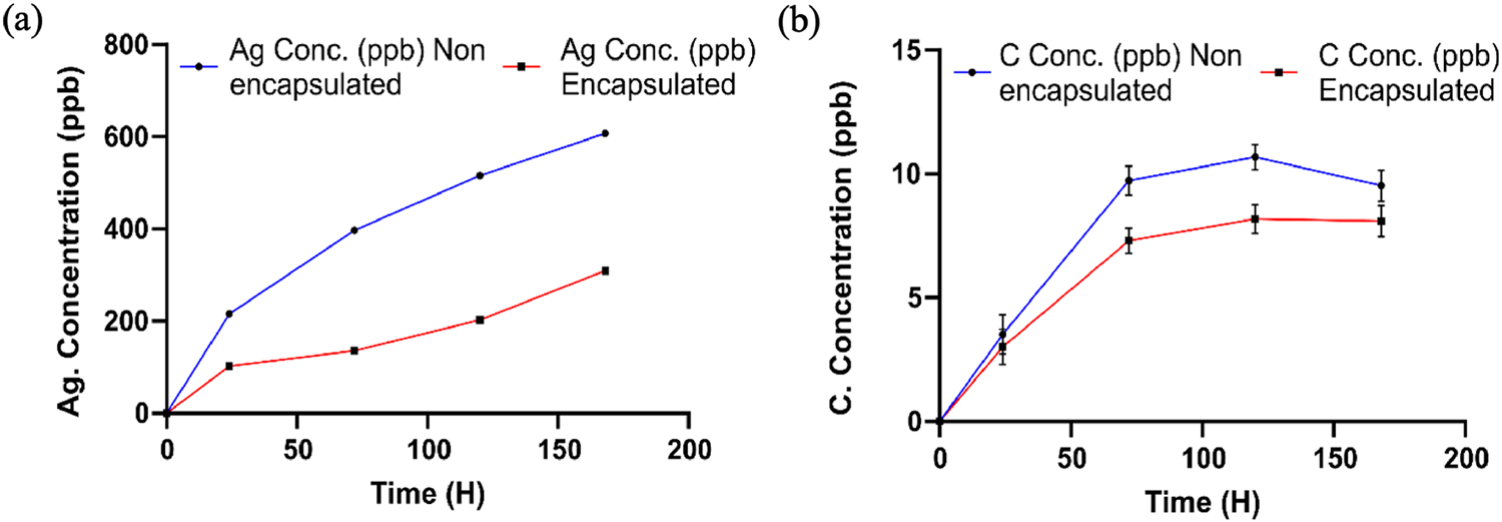
Quantitation of time-dependent leaching of (**a**) Silver (Ag^+^) ions, (**b**) Carbon atoms from the non-encapsulated (blue line) and encapsulated (red line) sensors at varying time points (1, 3, 5, and 7 days) using ICP-MS. The x-axis represents the time (H) and the y-axis represents the concentration of ions leached (ppb). All the data is depicted as mean ± SD from *n* = 3 independent experiments.

In our case the wearable device monitors uric acid levels as function of wound healing, where the sensors are used for extended periods (> 5 days). In such prolong use the screen-printed conductive inks may leach out toxic ions, potentially leading to adverse skin inflammation. Quantification of the ion dissolution and minimization of this leaching is therefore crucial. To analyze the leaching of carbon and Ag^+^ ions from the printed inks, the non-encapsulated and encapsulated sensors were incubated in de-ionized water for varying periods (up to 7 days) and quantified using inductively coupled plasma mass spectrometry (ICP-MS). Only trace levels (≤ 11 ppb) of carbon were detected for both the non-encapsulated and encapsulated sensors, indicating that negligible dissolution of carbon occurs (Fig. 2b). Alternatively, for both cases, a linear increase in Ag^+^ ion concentration was observed with time. After a 24 h incubation period, the concentration was 210 ppb and 100 ppb for the non-encapsulated and encapsulated sensor, respectively. The concentration of leached ions from the encapsulated sensors was seen to be ~ 2–3 times lower compared to the non-encapsulated sensors (Fig. 2a). While the extract incubated with a non-encapsulated sensor showed an Ag^+^ ion concentration of 607.31 ppb after a 7-day incubation period, the encapsulated sensor exhibited only a 309.19 ppb concentration. The mass spectrometric analysis confirmed that Ag^+^ ions leach out of printed inks and the polymeric encapsulation effectively inhibits the leaching.

### Exploring Ionic leaching from conductive inks and its correlation to cytotoxicity

**Figure 3:**
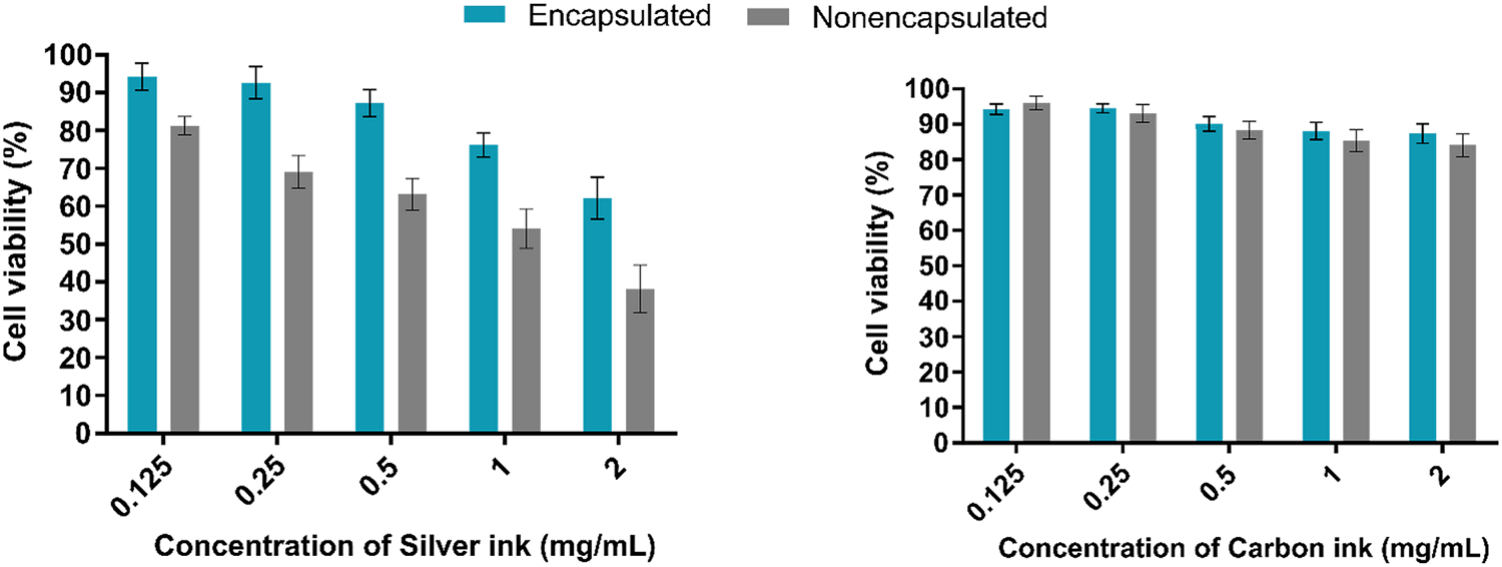
Toxicity assessment of the silver (left) and carbon inks (right) on the epidermal cell line HaCaT at varying concentrations. The x-axis represents the concentration of the ink (mg/ml) and the y-axis represents the cell viability (%). Bar graphs depict the cell viability assessed by MTT assay in response to the two inks after a 24 h exposure. All the data is depicted as mean ± SD from *n* = 3 independent experiments.

Assessing the cytotoxicity of particles or ions leaching from the inks in sensors placed in direct contact with the body is vital. The effect of silver ink on cell viability was expressed as a dose–response curve as shown in Fig. 3. A 24 h exposure to the non-encapsulated silver ink in the 0.125–2.0 mg ml^-1^ range resulted in a steep decrease in the cell viability (< 60% at higher concentrations), with the estimated 70% inhibitory concentration (IC70) being 0.25 ± 0.02 mg ml^-1^.

On the other hand, the encapsulated sensor showed only a marginal change. Cell viability after a 24 h exposure to inks at concentrations ≥ 0.5 mg ml^-1^ was > 90%, but exposure to higher concentrations exhibited viability in the 65–75% range. The higher cell viability in the case of the encapsulated sensor can be attributed to the hindered ionic (Ag^+^) leaching through the polymeric encapsulation layer. The results imply that encapsulation results in 22% reduced ionic leaching in turn leading to lower toxicity levels. Based on the above results, the IC70 concentration of the silver ink and the highest concentration of carbon ink was selected to further determine their toxicity pathway. To give a better understanding of sensor biocompatibility we further, evaluated the toxicity assessment of the encapsulated sensor in terms of ROS generation and caspase activation.The MTT assay showed that while carbon ink exhibited activity comparable to that of the control cells under all concentrations, the silver ink was cytotoxic (Fig. 3). Since the non-encapsulated carbon ink even at maximum concentrations (2 mg ml^-1^) caused a non-significant decrease in the cell viability (> 80%), carbon ink was excluded from further investigations to determine the toxicity pathway.

### Assessment of reactive oxygen species

**Figure 4:**
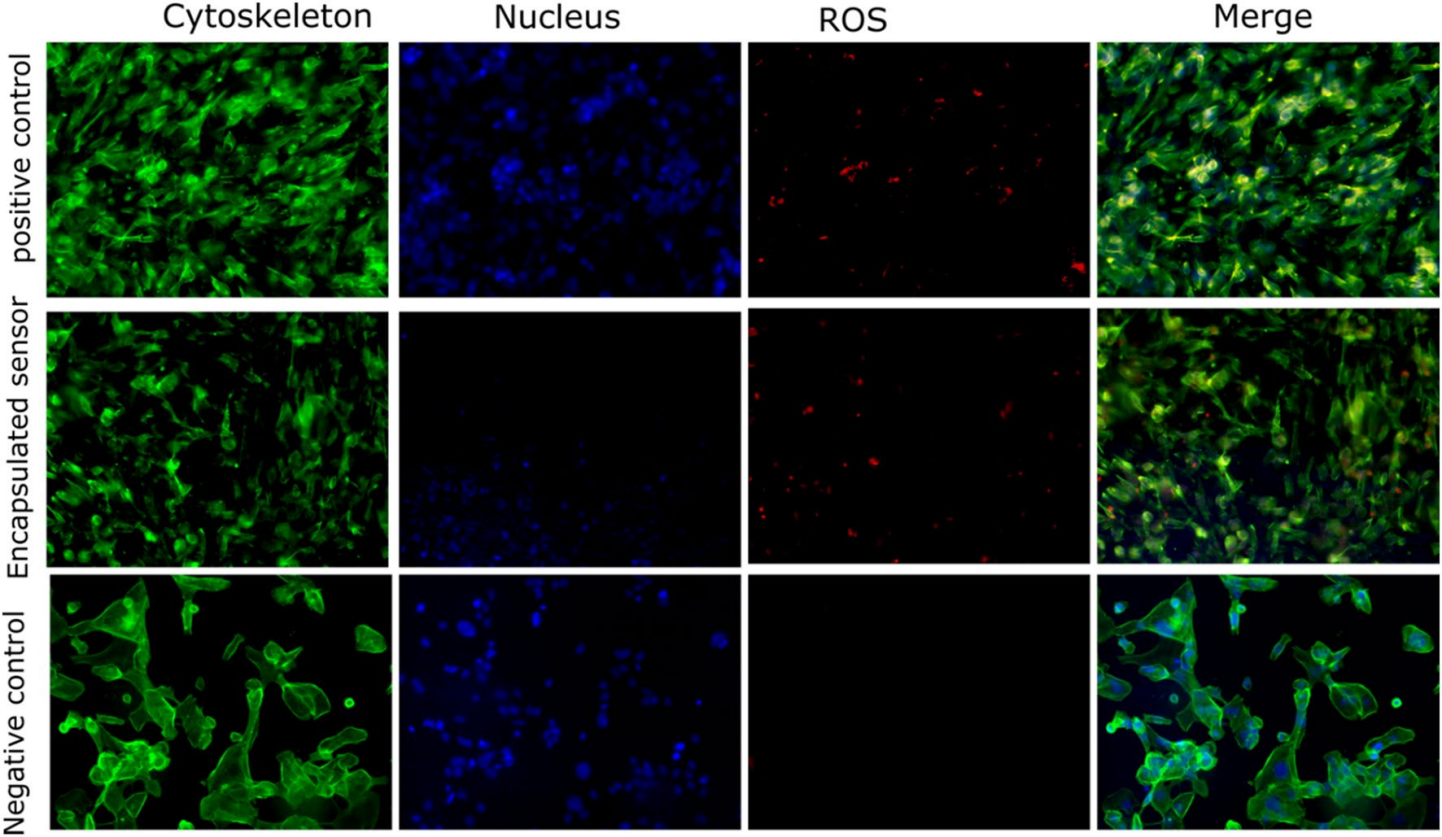
ROS generation assessment after a 24 h exposure to the different treatment groups on the epidermal cell line, HaCaT. Representative fluorescent images (magnification: 20x) captured after treatment with positive control (cells treated with 250 µM of hydrogen peroxide served as the positive control), an encapsulated sensor (encapsulated sensors fabricated using IC70 concentration of silver ink), and negative control (cells exposed to no treatment).

Reactive oxygen species are generated as by-products during mitochondrial electron transport or as intermediates of metal-catalyzed oxidation reactions. The sequential reduction of oxygen leads to the formation of ROS, which includes superoxide, hydrogen peroxide, hydroxyl ion, hydroxyl radical, and nitric oxide. An imbalance of ROS in the cells is termed oxidative stress. It can be seen from Fig. 4 that ROS generation is triggered when the epidermal cells are exposed to the encapsulated sensors. This is indicated by the red fluorescent cells shown in the images. Post 24 h we also observe that cells start to deform and detach suggesting ROS trigged cellular death. The results were indicative of the fact that although encapsulation prevents Ag^+^ ion leaching, it does not prevent inflammation. Therefore, more suitable encapsulation strategies or materials are needed for the development of future wearable sensors. To further investigate the pathway and evaluate the mode of cell death, we carried out a caspase assay.

### Assessment of cellular apoptosis

**Figure 5:**
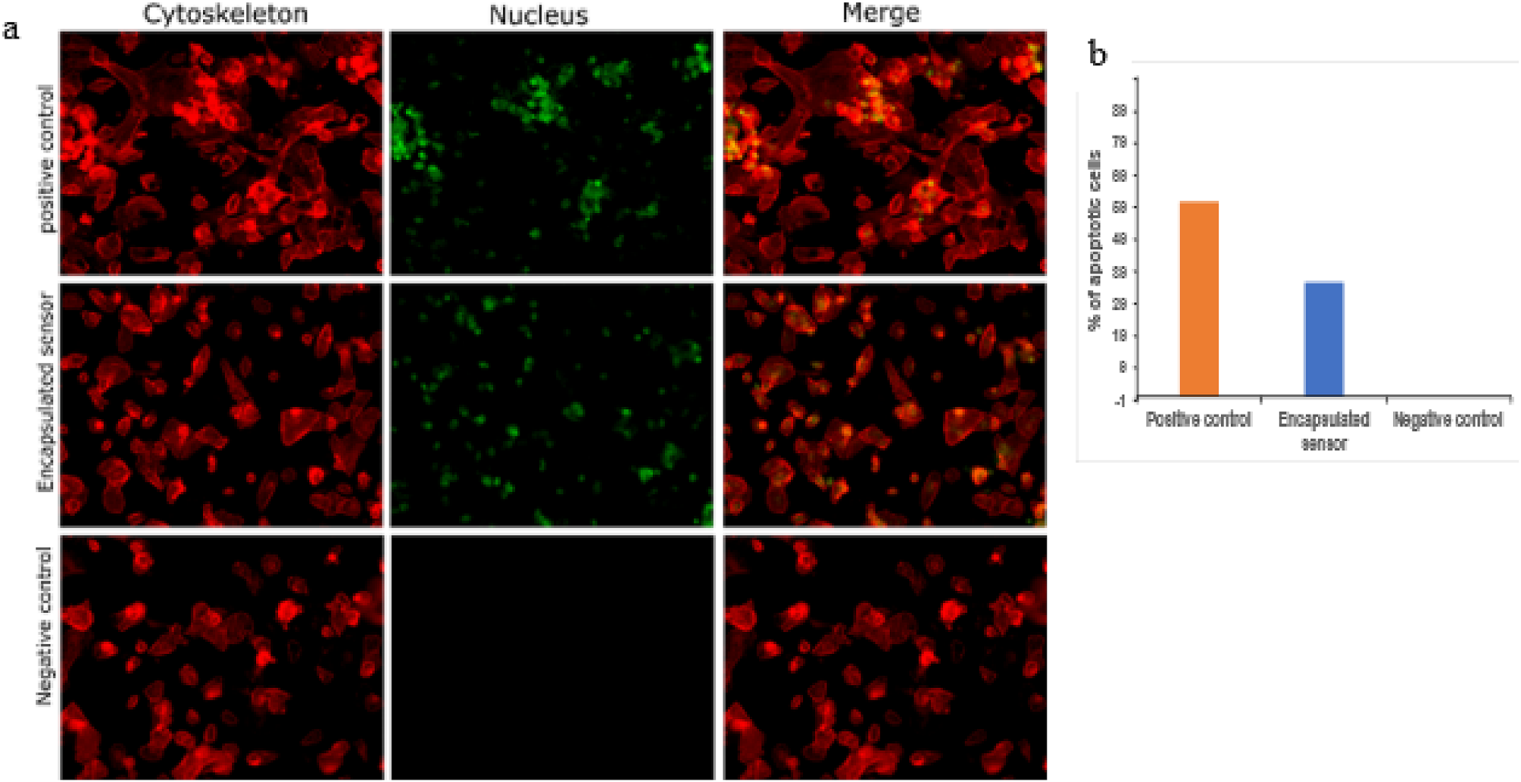
Caspase 3/7 activity assessment after a 24 h exposure to the different treatment groups on the epidermal cell line, HaCaT. (**a**) Representative fluorescent images (magnification: 20x) captured after treatment with positive control (cells treated with 10 µM of camptothecin for 12 h served as the positive control), an encapsulated sensor (encapsulated sensors fabricated using IC70 concentration of silver ink), and negative control (cells exposed to no treatment served as the negative control), (**b**) Quantitative representation of % of apoptotic cells after a 24 h exposure to the different treatment groups. The quantitation was performed using standard cell counting analysis of the obtained images (*n* = 5).

Caspase-3 and caspase-7, also known as executioner caspases, are major and early indicators of apoptosis. These caspases are activated following the leakage of cytochrome C from the mitochondria. Apoptotic cells were observed with Caspase 3–7 staining, with apoptotic cells stained as a bright green nucleus. The cells with a green nucleus in the merged images in Fig. 5 represent the apoptotic cells when subjected to the encapsulated sensors for 24 h. Post 24 h exposure the cells showed a positive caspase activity. As depicted in the figure, about 20% of the cells were apoptotic when subjected to sensors, indicating a high apoptosis-inducing ability. Our previous results had shown a high release of Ag^+^ ions from the sensors. The apoptotic results confirm that the Ag^+^ ions leached from the encapsulated inks induce cell suicide via the intrinsic pathway. A higher concentration of leached ions thus leads to a higher apoptotic activity. Cellular death is a slow process and the effect of sensor inducing cell death can only be observed in prolonged exposure to the sensors. In a use case such as wound monitoring, wherein the sensor may be placed for over 7 days on the wound bed^41,42^, the cumulative ionic leaching can elicit significant cellular death.

## Discussion

In summary, our results suggest that understanding the toxicity mechanism of ions leached from printed conductive inks is crucial for developing biocompatible sensors. The lack of literature on cytotoxic properties of printed conductive inks drove us to explore any possible ion leaching and their subsequent toxicity pathways. While previous efforts had focused on the toxicity of certain ions, the ion leaching from printed conductive inks had not yet been explored. This research characterized two widely used commercially available inks (silver and carbon) for any potential ionic leaching using ICPS analysis. Results suggest that non-encapsulated screen-printed silver inks lead to a high concentration of leached Ag^+^ ions (> 600 ppb) eliciting a highly toxic response (cell viability < 70%) for concentrations > 0.5 mg ml^-1^. This is concerning in cases where prolong exposure is mandatory for monitoring, which is not limited to wearables but also to implantable sensors. However, our study further shows that the leaching can be optimally reduced by designing the sensors to have an encapsulating layer. A 50% decrease in the ion concentration was observed when the inks were encapsulated with TPU polymer. The ratio of encapsulation directly influences on the flexibility of the sensors therefore there is a limitation on how much coating can be applied. In case of carbon leaching, it was observed to be minimal thus causing negligible cytotoxic response (cell viability > 80%). The toxicity pathway assessment results indicate that Ag^+^ ion toxicity is induced primarily through the apoptotic pathway. The ROS induction triggers cellular damage leading to apoptosis. This pathway can highlight some of the latest reports of allergic and inflammatory responses caused when wearing smartwatches (leaching of nickel). Our study thus indicates that identifying the leaching of a potential ion from a conductive ink or electrical components are critical in assessing and elicit a toxic response. Further there is a need towards development of sustainable encapsulating agents and flexible electronics which can significantly lower the inflammatory response. This study is expected to encourage the exploration of suitable conductive inks and explore the dynamic and interfacial interaction of the wearable sensors with cellular systems to guide the future development of biocompatible devices.

## Methods

### Fabrication of wearable electrochemical sensors

**Figure 6:**
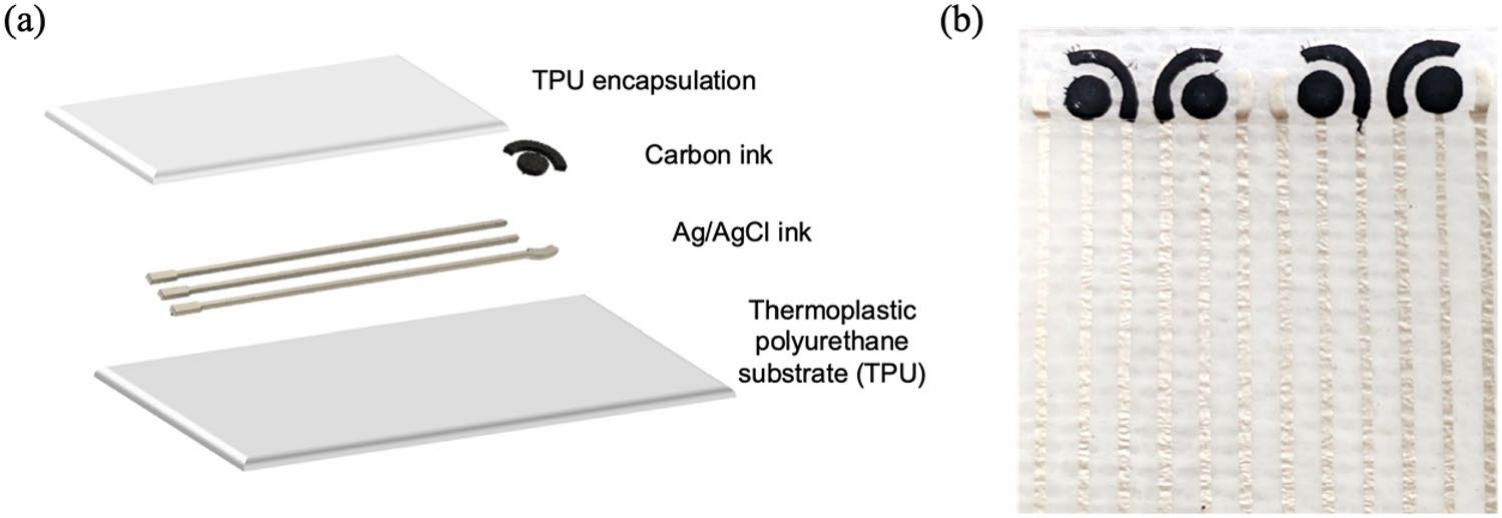
(**a**) Schematic representation of the different layers of the fabricated sensor used for detection of uric acid in chronic wounds. Sensors with and without the final TPU encapsulation were termed as encapsulated and non-encapsulated sensors respectively, (**b**) A photograph illustrating a prototype of a fabricated sensor array.

The wearable electrochemical sensors used for detection of uric acid to monitor wound healing was used in our study. The sensor was prepared in-house using a multilayered screen-printing approach. Conductive carbon (DuPont Intexar PE671) and silver/silver chloride (Ag/AgCl, Creative Materials, 127-48E) inks were sequentially printed onto a thermoplastic polyurethane (TPU, TE-11C DuPont) substrate to fabricate the three-electrode electrochemical sensors. The printing was performed using a hand-operated screen-printing setup and involved the following steps: (i) The electrode pattern mask was cut out on an adhesive vinyl using a Silhouette cameo 3 die cutter. (ii) The mask was carefully placed on a polyester screen (mesh size: 200). (iii) Ag/AgCl ink was transferred to the TPU substrate through a hand-held squeegee angled at 45°. (iv) The printed layer was cured for 15 min at 80 °C in a convection oven. (v) A layer of carbon ink was printed on top of the cured Ag/AgCl ink to fabricate the working and counter electrodes. (vi) The printed layer was cured for 15 min at 80 °C in a convection oven. These fabricated sensors were termed non-encapsulated sensors. An additional step was carried out for the fabrication of encapsulated sensors wherein the above-printed electrodes were further encapsulated with a TPU layer. A layer of TPU was heat pressed over the electrode traces leaving the working area and electrode pads exposed. These electrodes are hereafter referred to as encapsulated sensors. An exploded view of the different sensor layers and a photograph of a sensor array is shown in Fig. 6a and b respectively.

### Cell culture

The keratinocyte cell line (HaCaT) was provided by Dr. Marcus Cooke at the FIU Department of Environmental Health Sciences, Miami, Florida. The cell line was cultured and maintained in Dulbecco's Modified Eagle's Medium (DMEM, Gibco, USA) supplemented with 10% fetal bovine serum (FBS, Gibco, USA) and 1% antibiotics (penicillin/streptomycin, Gibco, USA). The cell line was maintained in an atmosphere of 5% CO_2_ at 37 °C with 70% humidity.

### Time-dependent quantitation of ionic leaching

Preventing direct exposure to metallic ions on the skin is important to prevent any cell impairment. We hypothesized that the inks used (0.5 µg ml^-1^) in sensor fabrication may potentially result in the leaching of metal ions. To examine the leaching of Ag^+^ ions and carbon atoms from the printed Ag/AgCl and carbon inks respectively, the encapsulated and non-encapsulated sensors were incubated in 10 ml of deionized water for 1, 3, 5, and 7 days. The Ag^+^ ion dissolution was analyzed using inductively coupled plasma mass spectrometry (Thermo iCAP TQ triple Quadrupole) and the total dissolved carbon content (C) was measured using a total organic carbon analyzer (Shimazu TOC-V Analyzer). All the measured values were read against a calibration curve obtained from a set of standards.

### Assessment of correlation of ionic leaching to cytotoxicity

The cell viability after exposure to the printed conductive inks at different ink concentrations was probed using the 3-(4, 5 dimethyl thiazolyl-2)-2, 5-diphenyltetrazolium bromide (MTT, Sigma-Aldrich, USA) assay. HaCaT cells were cultured up to a confluence of 75% and then trypsinized using a 0.25% trypsin solution (Gibco, USA). Cells were harvested and counted using a hemocytometer, and approximately 5000 cells/well were inoculated in a 96-well plate. The plate was incubated at 37ºC in 5% CO_2_ for 24 h, following which the cells were subjected to varying concentrations of the conductive inks (0.125 – 2 mg ml^-1^) for 24 h. Cells not exposed to any ink were used as the negative control. After the exposure, 10 µL of MTT reagent (15 µg ml^-1^) prepared in 1 × phosphate-buffered solution (PBS) (Gibco, USA) was added to each well. The plate was then incubated at 37 °C for 4 h, followed by the addition of 200 µL of dimethyl sulfoxide (DMSO, Sigma-Aldrich, USA) to each well. DMSO aids in dissolving the formazan crystals resulting in purple color. The cell viability was assessed by measuring the absorbance at 570 nm using a plate reader (ThermoFisher Sc. Multiskan™ FC microplate reader).

### Assessment of toxicity mechanism via early generation of reactive oxygen species (ROS)

The total ROS generation in the cells on exposure to the sensors was assessed using the ROS generation Assay Kit, ThermoFisher. HaCaT cells were cultured up to a confluence of 75% and then trypsinized using a 0.25% trypsin solution (Gibco, USA). Cells were harvested and counted using a hemocytometer, and approximately 5000 cells/well were inoculated in a 96-well plate. The plate was incubated at 37ºC in 5% CO_2_ for 24 h, following which the cells were subjected to the encapsulated and non-encapsulated sensors for 24 h. The exposed cells were then fixed with 4% paraformaldehyde, washed with 1 × PBS, and permeabilized using 0.1% Triton-X. After washing, the cells were stained with rhodamine phalloidin for cytoskeleton staining and Hoechst 33,342 for nuclear staining. Cells were imaged at 20 × using a Axio Scope.A1 fluorescence microscope (Zeiss, USA).

### Assessment of molecular mode of cell death

Cellular apoptosis on exposure to the sensors was assessed by monitoring the activation of Caspase-3/7. The Caspase-3/7 assay (CellEvent™ Caspase-3/7 Green Detection Reagent, ThermoFisher) consists of a fluorogenic substrate, that has a four amino acid peptide (DEVD) conjugated to a DNA binding dye. On activation of Caspase-3/7 in the apoptotic cell, the DEVD peptide is cleaved producing 6-aminoluciferin, and the dye binds to DNA, producing a bright fluorescence response. The intensity of the response is proportional to the amount of Caspase-3/7 activity. The assay is highly specific for caspase-3/7 activation and can be used to monitor their activation with live-cell fluorescence imaging. Since the cleaved reagent labels the nuclei of caspase 3/7–positive cells, the stain can be used to assess the nuclear morphology, including condensed nuclei which is typical of late-stage apoptosis. HaCaT cells were cultured up to a confluence of 75% and then trypsinized using 0.25% trypsin. Cells were harvested and counted using a hemocytometer, and approximately 5000 cells/well were inoculated in a 96-well plate, followed by exposure to the encapsulated and non-encapsulated sensors for 24 h. Subsequently, 100 μL of Caspase-3/7 reagent (5 μM) was added to each well. After incubation for 30 min at 37 °C, the cells were fixed with 4% paraformaldehyde, washed with 1 × PBS, and permeabilized using 0.1% Triton-X. Fluorescence images were captured at 20 × using a Axio Scope.A1 microscope (Zeiss, USA).

### Statistical analysis

All the experiments were carried out in triplicate (*n* = 3) and all the values are represented as mean ± standard deviation (SD). Statistical analysis was carried out using the GraphPad Prism program (GraphPad Software, USA).

## Data Availability

All data generated or analyzed during this study are included in this published article.
